# Can we change the way we look at BCG vaccine?

**DOI:** 10.4103/1817-1737.49419

**Published:** 2009

**Authors:** Sahal Abdulaziz Al-Hajoj

**Affiliations:** *TB Research Unit, Comparative Medicine, King Faisal Specialist Hospital and Research Center, P. O. Box 3354, Riyadh 11211, Saudi Arabia. E-mail: hajoj@kfshrc.edu.sa*

Sir,

I have read with great interest the review article entitled “Diagnosis of latent tuberculosis can we do better by Dr. Ibrahim O. Al-Orainy; therefore, I would like to shed some light on the topic and give my comments thereon.[1] Tuberculosis is a major health threat in Saudi Arabia, as well as anywhere in the world. Saudi Arabia is a special place as it has 6 million expatriates for work purposes. In addition, 2-3 million visitors yearly do visit the country for religious rituals. Recent findings showed that Saudi Arabia harbors many different clades (families), which are imported strains. One of those clades is the Beijing family, a strain notorious for its easy transmissibility and drug resistance. Also, the data shows that we do have ongoing active transmission — as it is identified by the high rate of clustering, a tool which is used by epidemiologists to identify ongoing and recent transmission. In addition to the imported strains, the country has its own endogenous strains, which also are involved in active transmission.[2]

Recently, King Faisal Specialist Hospital and Research Centre held a conference on “Infections in Immunocompromised host” (18-19 November 2008). In this symposium, many specialists expressed their reservations about administering tuberculosis vaccine (BCG vaccine) to newborn children. They pointed out that in some cases of pediatrics with immune deficiencies, it is very important to delay the vaccination plan until the possibility of genetic immune deficiency has been ruled out. This is due to of the complication of the vaccine and the dissemination of the disease.[3]

In addition, the country lacks data regarding the efficiency of BCG vaccination. In other words, we do not know whether BCG vaccine is protective in our community or not.

The other thought is to stop altogether the BCG vaccination. The philosophy behind this thought is that BCG vaccine does not protect against TB; otherwise, why do we have up to 4000 cases yearly (according to a recent report by the Ministry of Health) despite the fact that all our babies are vaccinated at birth? Tuberculosis is on the rise, according to a recent report by the Ministry of Health. The report showed that the number of cases for the year 2007 was indeed higher than the number of cases reported in the year 2006. BCG vaccination is a big problem, especially in an environment such as Saudi Arabia, where there is an ongoing transmission of tuberculosis, as well as infection with mycobacterium other than tuberculosis. The positivity of skin test means i) exposure to real infection, ii) exposure to environmental mycobacterium or iii) reaction due to BCG vaccine itself. In an environment such as Saudi Arabia, interpretation of skin test is extremely difficult in the light of the fact that the protein used in skin test is shared by many species of mycobacterium. Shared protein is the main cause of the confusion.[4] It is for this reason that the BCG vaccine is not given in many countries such as Netherlands, UK and, recently, France; yet these countries have better control over tuberculosis as compared to many countries where BCG vaccine is mandatory, including Saudi Arabia.

With regards to the diagnostic tools for latent tuberculosis mentioned by Dr. Al-Orainy, we [Tuberculosis Research Unit, Department of Comparative Medicine, King Faisal Specialist Hospital and Research Centre] would like to announce the availability of whole blood interferon gamma testing. We used the test on different types of patients so far, and the results are promising. [Unfortunately at this stage, before the completion of the project, we cannot disclose such results].

Interferon-γ tests (interferon gamma release assays, IGRAs) are based on the ability of the *Mycobacterium tuberculosis* antigens for early secretory antigen target 6 (ESAT-6) and culture filtrate protein 10 (CFP-10) to stimulate host production of interferon-gamma. Because these antigens are not present in nontuberculous mycobacteria or in BCG vaccine, these tests can distinguish between latent tuberculosis infection in asymptomatic patients and exposure to BCG or nontuberculous mycobacteria. The test is approved for diagnosis of latent tuberculosis and has also been used in patients with pulmonary tuberculosis.[5]

We fully approve of the establishment of such diagnostic tools everywhere in the country. It is an expensive test at this particular stage but it is worth using, as delay in diagnosing difficult cases such as extrapulmonary is more costly to the patients (as it might cost them their lives). Usually, treating dormant tuberculosis, particularly when it comes to close contact individual, is far cheaper than treating patients after they show full symptoms of the disease. On the other hand, it is a very bad practice to give prophylaxis to treat dormant tuberculosis based on skin test results as the test has proved its inability to distinguish real infection from exposure to environment tuberculosis or BCG vaccine. Also, giving a prophylaxis indiscriminately based on skin test gives a chance for drug resistance to develop.

In conclusion, we think it is the right time now to review our policy of giving BCG vaccine to our newly born babies as it is creating more confusion rather than providing protection: It causes diseases in immunocompromised patients. BCG vaccine causes confusion when it comes to interpretation of skin test results. Also, it gives false-positive and false-negative results. We are aware of what is said in literature — that BCG vaccine prevents miliary tuberculosis — but it has no evidence based on the actual situation in our society. In addition, we need to improve the case finding, improve the treatment outcomes and break the cycle of ongoing transmission in order to control tuberculosis infection in Saudi Arabia.
